# Sex Differences in Immunology: More Severe Development of Experimental Pulmonary Hypertension in Male Rats Exposed to Vascular Endothelial Growth Factor Receptor Blockade

**DOI:** 10.1155/2015/765292

**Published:** 2015-09-01

**Authors:** Julien Guihaire, Tobias Deuse, Dong Wang, Elie Fadel, Hermann Reichenspurner, Sonja Schrepfer

**Affiliations:** ^1^TSI Laboratory, University Heart Center Hamburg, 20246 Hamburg, Germany; ^2^Cardiovascular Research Center Hamburg (CVRC) and DZHK (German Center for Cardiovascular Research), Partner Site Hamburg/Kiel/Lübeck, 20246 Hamburg, Germany; ^3^Cardiovascular Surgery, University Heart Center Hamburg, 20246 Hamburg, Germany; ^4^Thoracic and Vascular Surgery and Heart-Lung Transplantation, Marie Lannelongue Hospital, University of Paris-Sud, 92350 Le Plessis-Robinson, France; ^5^Department of Cardiothoracic Surgery and Stanford Cardiovascular Institute, Stanford University, Stanford, CA 94305, USA

## Abstract

*Background.* The epidemiology of pulmonary hypertension (PH) is characterized by a female preponderance, whereas males share higher severity of the disease. *Objective.* To compare the severity of experimental PH between male and female athymic rats. *Methods.* PH was induced in 11 male and 11 female athymic rats (resp., SU_M and SU_F groups) using an inhibitor of VEGF-receptors I and II, semaxanib (40 mg/kg). After 28 days, right ventricular (RV) remodeling, systolic function, and hemodynamics were measured using echocardiography and a pressure-volume admittance catheter. Morphometric analyses of lung vasculature and RV myocardium were performed. *Results.* Four weeks after semaxanib injection, RV end-systolic pressure was higher in SU_M than in SU_F. Males developed marked RV enlargement and systolic dysfunction compared to females. Impairment of RV-PA coupling efficiency was observed only in SU_M. The smooth muscle cells of the pulmonary arteries switched from a contractile state to a dedifferentiated state only in males. *Conclusions.* Female athymic rats were protected against the development of severe PH. RV-PA coupling was preserved in females through limitation of pulmonary artery muscularization. Control of smooth muscle cells plasticity may be a promising therapeutic approach to reverse established vascular remodeling in PH patients.

## 1. Introduction

Pulmonary hypertension (PH) is a disabling disease characterized by higher prevalence in females [1]. Considering the independent predictors of mortality, male gender is however one of the strongest [2]. Since few males are included in clinical trials investigating PH, there is lack of data regarding the precise role of estrogens in the development and progression of pulmonary vascular disorders. Experimentally, estrogens exhibit protective effects on the pulmonary vasculature in classical models of PH in rodents including the chronic hypoxia and monocrotaline models [3, 4]. Sweeney et al. suggested in 2009 that female gender may protect against semaxanib/hypoxia related angioproliferative PH, possibly by preventing semaxanib-induced pulmonary endothelial apoptosis [5]. In the present study, we induced PH in male and female rats to investigate the influence of gender difference on the development and severity of PH. We did not use the classical model of angioproliferative PH which combines semaxanib with chronic hypoxia because of the lack of hypoxic chambers in our animal facilities. However we injected the double dose of semaxanib in athymic nude rats since T-cell deficiency has been demonstrated to increase semaxanib toxicity in the pulmonary vasculature [6].

## 2. Methods

Thirty eight 6-week-old athymic RNU-rats (Crl:NIH-*Foxn1* ^*rnu*^) rats including 19 males and 19 females were purchased (Charles River, Sulzfeld, Germany). The mean body weight was 213 ± 67 gr. The research protocol was approved by our Institutional Committee on Animal Welfare. Experimental PAH was induced using the tyrosine kinase inhibitor semaxanib (SU5416, SUGEN Inc., Sigma-Aldrich, Saint Louis, MO) suspended in CMC (0.5% (w/v) carboxymethylcellulose sodium, 0.9% (w/v) sodium chloride, 0.4% (v/v) polysorbate 80, and 0.9% (v/v) benzyl alcohol in deionized water) [4]. Eleven males (SU_M group) and 11 females (SU_F group) received a single subcutaneous injection of semaxanib (40 mg/kg;), while 8 males (RNU_M group) and 8 females (RNU_F group) only received an injection of CMC vehicle. Animals received humane care in compliance with the “Principles of Laboratory Animal Care” formulated by the National Society for Medical Research and the “Guide for the Care and Use of Laboratory Animals” prepared by the Institute of Laboratory Animal Resources and published by the National Institutes of Health (NIH Publication number 86-23, revised 1996) and were housed under normoxic conditions (animal facilities of the University Hospital Eppendorf, Hamburg, Germany).

**Figure 1:**
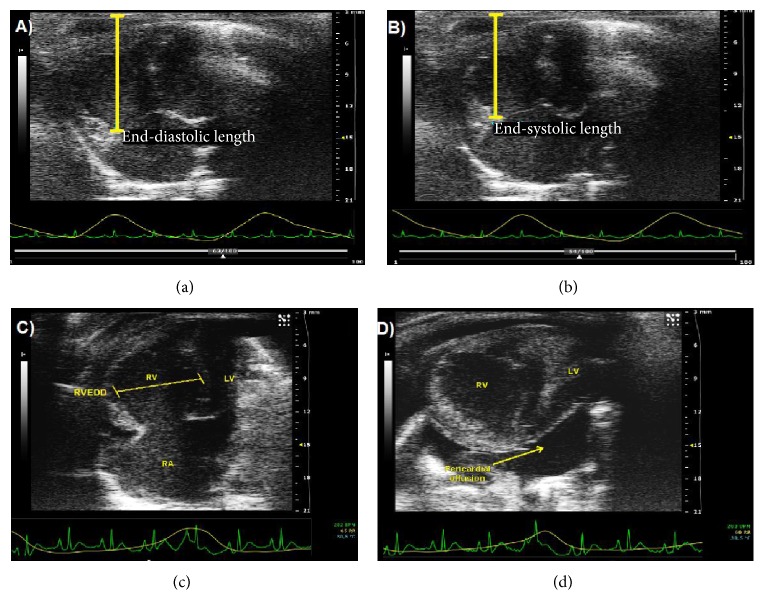
Representative cardiac echo imaging in rats at 3 weeks. The tricuspid annular plane systolic excursion (TAPSE) is measured on a 4-chamber apical view (2D-mode) as the difference in length between the tricuspid annulus and a fixed point at the end-diastolic (a) and end-systolic times (b). On the same view, the right ventricular end-diastolic diameter (RVEDD) is measured transversally from the midinterventricular septum to the mid-right-ventricular free wall (c, 2D-mode). In SU_M at 3 weeks, pericardial effusion can be observed (d, parasternal short axis, 2D-mode). RV: right ventricle; RA: right atrium; LV: left ventricle.

**Figure 2:**
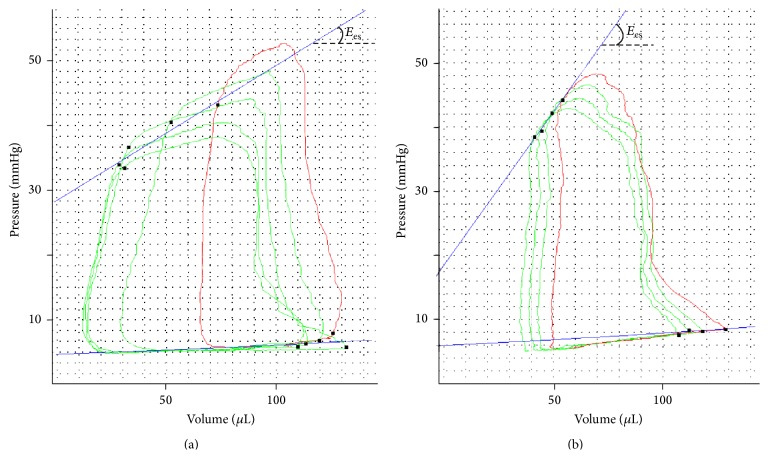
Representative pressure-volume loops from SU_M and RNU_M at 4 weeks. At baseline, the pressure-volume loop is in red. During occlusion of the inferior vena cava, the right ventricular end-diastolic volume decreases, shifting the following loops (green) toward the left. Similarly the right ventricular end-systolic pressure decreases, shifting the loops to the bottom. The linear end-systolic relationship is represented in blue, linking all end-systolic points from the successive pressure-volume loops during transient occlusion of the inferior vena cava. The slope of this line defines the end-systolic elastance of the ventricle (***E*_es_**).

All procedures were performed under general anesthesia combining buprenorphine (0.5 mg/kg, SC) with inhaled isoflurane (2% mixed with 0.5 L/min 100% oxygen for induction, 1% to 1.5% for maintenance). Rats were then placed in the supine position (head up) onto a warmed plate to help maintain the rat core temperature at 37°C.Cardiac echography (Vevo 2100 system, VisualSonics, Toronto, Canada) was performed 21 days after PH induction to assess the mid-right-ventricular end-diastolic diameter (RVEDD) and the tricuspid annular plane systolic excursion (TAPSE). The ultrasound transducer (13–24 MHz, MS250, VisualSonics) was manually immobilized on the shaved area overlying the heart with a 45° angle. A complete two-dimensional (2D) 4-chamber view was obtained by adjusting the position of the probe parasternally over the cardiac apex. TAPSE was measured as the maximal motion toward the apex of the lateral tricuspid annulus (Figure 1). A 1.2 Fr–4.5 mm electrode spacing admittance catheter (Scisence, Ontario, Canada) was introduced in the right ventricle through the apex via an open-chest approach at day 28. The probe was connected to the ADVantage admittance pressure-volume system (Scisence) for real-time assessment of absolute right ventricular (RV) volume and pressure. Pressure-volume loops were recorded under general anesthesia (inhaled isoflurane 2% and buprenorphine 0.5 mg/kg, SC) and the RV end-systolic pressure-volume relationship was assessed during transient external occlusion of the inferior vena cava using nontoothed DeBakey forceps. TheRV end-systolic elastance (***E*_es_**) was defined as the slope of the end-systolic pressure-volume relationship (Figure 2). The pulmonary arterial elastance (***E*_a_**) was defined as follows: RV end-systolic pressure/RV stroke volume. Right ventricular-pulmonary arterial (RV-PA) coupling efficiency was quantified by the ***E*_es_**/***E*_a_** ratio.

Animals were sacrificed at day 28 for heart and lung procurement. To assess RV hypertrophy, the Fulton index was calculated as the weight ratio of the right ventricle and left ventricle + septum. After one-day fixation in 4% buffer formalin solution, 5 *μ*m sections were performed on paraffin-embedded tissues. Medial thickness of the pulmonary arteries (50 to 100 *μ*m of maximal diameter adjacent to bronchioles) was calculated as the percentage of medial layer as compared with the cross-sectional diameter of the artery from one external elastic lamina to the opposed external elastic lamina (Elastica von Gieson staining; Thermo Fisher Scientific, Schwerte, Germany; magnification ×400) [7]. To quantify RV hypertrophy, the cross-sectional area of 20 randomized cardiomyocytes was measured in the subepicardial layer of the RV free wall (Hematoxylin-eosin staining; Sigma-Aldrich; magnification ×400).

To study gender differences in smooth muscle cells (SMCs) plasticity, paraffin-embedded lungs underwent heat-induced antigen retrieval with Dako antigen retrieval solution (Dako, Glostrup, Denmark) in a steamer for 20 min. After blocking with Image-iT FX signal enhancer (Invitrogen, Carlsbad, Ca) for 30 minutes, slides were incubated with primary antibodies against smooth-muscle-heavy-chain (SM heavy chain; EPR5335, Abcam, Cambridge, UK) and embryonic smooth muscle myosin heavy chain (SMemb; 3H2, Yamasa, Tokyo, Japan) for 16 h at 4°C. After washing with PBS, sections were incubated for 1 h at 37°C with the corresponding secondary antibody conjugated with Alexa Fluor 488 or Alexa Fluor 555 (Invitrogen). DAPI was used to counterstain cell nuclei. Images were obtained by a Nikon Eclipse TiE microscope (Nikon, Tokyo, Japan) equipped with the Perkin Elmer UltraVIEW VoX confocal imaging system (Perkin Elmer, Waltham, MA).

All variables were reported as mean value ± standard deviation. One-way analysis of variance was performed for between-group comparisons with a 95% confidence interval using the Prism software (GraphPad, La Jolla, CA). Only animals who survived until day 28 were included in the statistical analysis.

## 3. Results

**Figure 3:**
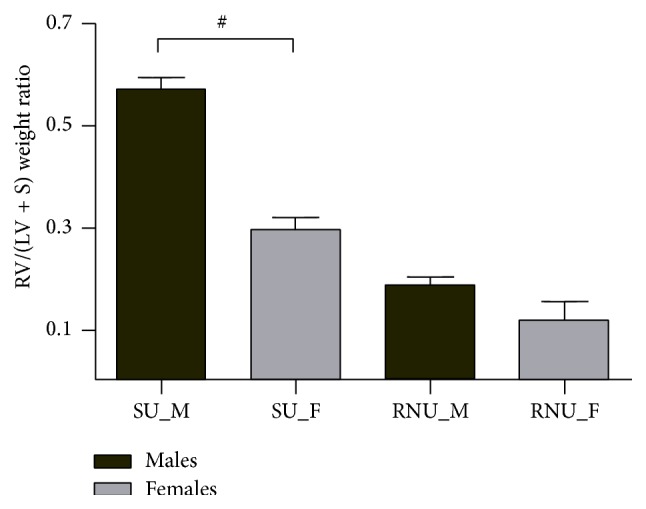
Fulton index (RV/(LV + S)) in rats at 4 weeks. The right ventricle of Su_M was significantly hypertrophied compared to Su_F, RNU_F, and RNU_M, as illustrated by the elevated Fulton index in this group. # explains *P* < 0.001.

**Figure 4:**
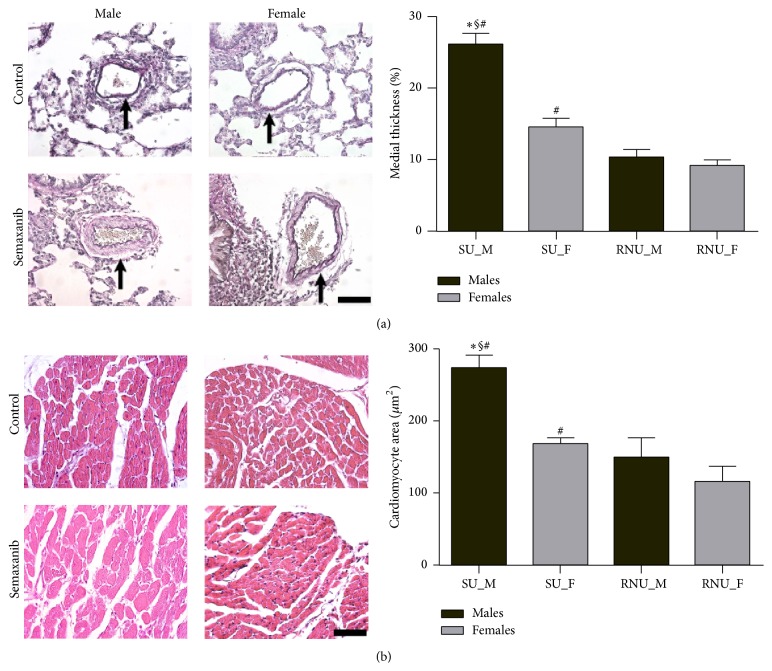
Morphometry of the pulmonary arteries and of the right ventricle at 4 weeks. (a) Pulmonary artery muscularization, defined by medial thickening over 10% of the cross-sectional diameter, was remarkable in male rats, while females did not develop significant medial hypertrophy in response to semaxanib injection (black arrows show the medial layer). (b) Similarly, cardiomyocytes hypertrophy was lower among females exposed to experimental PAH. Scale bars represent 50 *μ*m. Abbreviations are in the text. *∗*  § and # explain *P* < 0.05, respectively, compared to SU_F, RNU_M, and RNU_F (one-way ANOVA).

**Figure 5:**
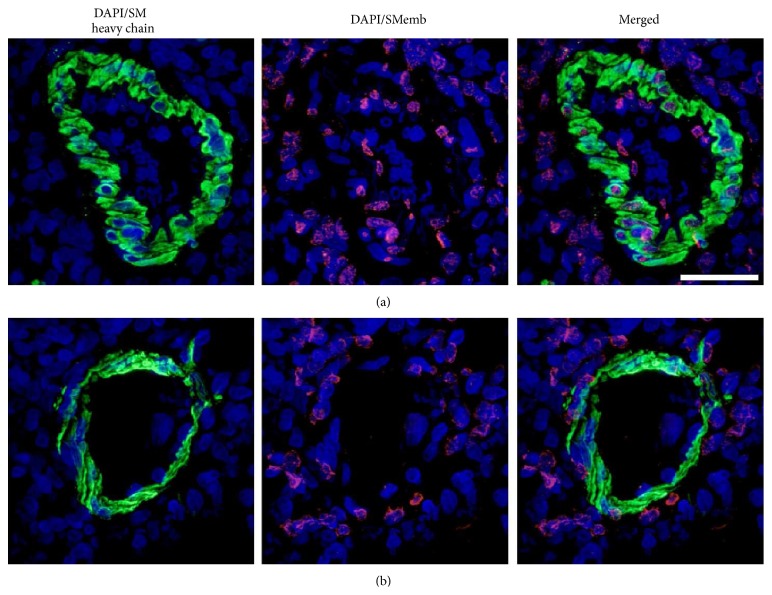
Immunofluorescence analysis of the pulmonary arteries at 4 weeks. (a) In male rats, SMemb, the embryonic form of smooth muscle myosin heavy chain and a marker for dedifferentiated SMCs, was increased within the pulmonary arteries, indicating SU-induced SMC-phenotype switch. (b) In contrast, confocal immunofluorescence revealed differentiated SMC-phenotype in female pulmonary arteries. (Blue: nucleus staining with DAPI; green: smooth-muscle-heavy-chain (SM heavy chain); pink: embryonic smooth muscle myosin heavy chain (SMemb); scale bars represent 33 *μ*m).

Among the 11 semaxanib-treated males, 3 died before day 28 (on days 21, 21, and 26) and 3 developed marked pleuropericardial effusion. All females survived until day 28 without any thoracic effusion. Induction of experimental PH resulted in a significant enlargement of the right ventricle after 3 weeks in males compared to females (RVEDD: 5.6 ± 0.7 versus 2.5 ± 0.4 mm, *P* < 0.01). In addition, TAPSE was lower in the SU_M group (1.28 ± 0.15 versus 2.09 ± 0.35 mm, *P* < 0.01). Hemodynamic parameters after 28 days are listed in Table 1. In response to semaxanib injection, athymic males showed significant RV pressure overload during systole, with an average of 66.3 mm Hg. Increased ***E*_a_** combined to altered ***E*_es_** in male rats resulted in impairment of RV-PA coupling efficiency, while ventricular-arterial coupling was preserved in the SU_F group. Morphometric analysis of the lung vasculature showed a very small rate of intimal remodeling and plexiform lesions were not remarkable among semaxanib-treated animals. Hypertrophy of the pulmonary medial layer was significantly higher in SU_M group (25.9% ± 4.5% versus 14.6% ± 2.8%, *P* < 0.001). RV pressure overload was associated with RV hypertrophy as illustrated by the higher Fulton ratio in SU_M compared to SU_F (0.57 ± 0.07 versus 0.29 ± 0.07, *P* < 0.001; Figure 3). Similarly cardiomyocytes hypertrophy was only observed in athymic males (273 ± 48 versus 168 ± 24 *μ*m^2^, *P* < 0.001) (Figure 4). Both Fulton index and cardiomyocytes area were not significantly different between RNU_M and RNU_F. The SMC-phenotype switch in pulmonary arteries from a contractile (SM heavy chain-positive) to a dedifferentiated (SMemb-positive) state after induction of PH was only observed in male rats (Figure 5).

## 4. Discussion

Athymic female rats developed less severe PH in response to vascular endothelial growth factor (VEGF) receptor blockade. As a consequence, RV function and RV-PA coupling were preserved only in females in this experimental descriptive study. We hypothesize a protective role of endogenous estrogens against PH in semaxanib-treated rats under normoxic conditions. Our findings are in accordance with previous experimental studies showing that ovariectomized rats develop more severe PH in response to chronic hypoxia or to monocrotaline [3].* In vitro*, estrogens effects on the pulmonary circulation include increase in prostacyclin release, upregulation of the endothelial nitric oxide synthase, and downregulation of endothelin-1 expression [8]. However, the effects of steroid hormones and their analogues in PH patients are controversial [9]. Estrogens exhibit proangiogenic properties through the upregulation of VEGF receptors. Since VEGF is a key regulator of endothelial cell survival in the lung, estrogen-therapy may interfere with the development of the pulmonary vascular lesions in PH patients [10]. Protective effects of estrogens might thus concern hyperproliferative SMCs rather than phenotypically altered endothelial cells. This is in agreement with the present results, since isolated hypertrophy of the medial layer was the hallmark of PA narrowing in our athymic rat model. Since we used a modified model of angioproliferative PH combining semaxanib injection in athymic nude rats under normoxic conditions [6], animals were thus exposed to a strong angioproliferative stress resulting in impairment of ventricular-arterial coupling efficiency, pleuropericardial effusion, and sudden death in males. Taraseviciene-Stewart et al. reported that T-cell deficiency is associated with severe PH after semaxanib injection under normoxic conditions [6]. In this modified angioproliferative PH model, animals share a progressive RV remodeling and dysfunction, with various ranges of severity, which was relevant for between-group comparisons.

Effects of estrogens on the pulmonary vasculature are complex and not clearly understood. Sex differences have been poorly investigated in PH. Jacobs et al. recently reported in a retrospective clinical study worse outcome in men, suggesting gender difference in cardiac adaptation to chronic PH [11]. The present study shows that female rats are experimentally protected against the development of severe PH and that the phenotype switch of SMCs from contractile to a dedifferentiated state occurs only in male rats after PH induction. The lack of major intimal damage in our model suggests that endogenous estrogens preserved RV-PA coupling through limitation of medial muscular thickening. We did not study ovariectomized females to prove whether PH severity was only related to the absence of estrogens in males or whether the gender differences observed here were only determined by a genetic predisposition between male and female rats. Further studies are necessary to investigate the potential of estrogen-therapy to reverse established pulmonary vascular remodeling in PH patients.

## 5. Conclusions

Female athymic rats developed less severe pulmonary vascular damage as males in response to angioproliferative stress. Moderate pulmonary artery muscularization and the lack of phenotype switch of smooth muscle cells from contractile to dedifferentiated state was associated with preserved ventricular-arterial coupling and RV efficiency in semaxanib-treated females. Control of smooth muscle cells plasticity may be a promising therapeutic approach to reverse established vascular remodeling in PH patients.

## Figures and Tables

**Table 1:** Right heart hemodynamics at 4 weeks.

Group (*n*)	RNU_M (8)	RNU_F (8)	SU_M (8)	SU_F (11)
Semaxanib (mg/kg)	0	0	40	40
Heart rate (beat/min)	269 ± 37	311 ± 35	296 ± 48	280 ± 73
RVESP (mm Hg)	21 ± 2.5	24.3 ± 8.1	66.3 ± 8.9^*∗*#§^	29.5 ± 13.4
RVEDP (mm Hg)	1.5 ± 3.5	3 ± 3.6	6.2 ± 2.8^§^	2.1 ± 4.3
CO (mL/min)	134 ± 4	122 ± 5	58 ± 24^*∗*#§^	89 ± 17
PVR (dyn·s·cm^−5^)	0.013 ± 0.002	0.016 ± 0.006	0.103 ± 0.06^*∗*#§^	0.027 ± 0.01
*dP*/*dt* max (mm Hg/s)	1173 ± 161	1311 ± 414	2636 ± 495^*∗*#§^	1374 ± 320
*dP*/*dt* min (mm Hg/s)	−1318 ± 37	−871 ± 232	−2370 ± 912^*∗*#§^	−1072 ± 415
*E*_es_ (mm Hg/*µ*L)	0.19 ± 0.05	0.21 ± 0.15	0.12 ± 0.06^*∗*#§^	0.18 ± 0.05
*E*_a_ (mm Hg/*µ*L)	0.04 ± 0.01	0.06 ± 0.03	0.33 ± 0.12^*∗*#§^	0.09 ± 0.02
*E*_es_/*E*_a_	4.75 ± 0.63	3.5 ± 0.45	0.36 ± 0.17^*∗*#§^	2.1 ± 0.34

RVESP: right ventricular end-systolic pressure; RVEDP: right ventricular end-diastolic pressure; CO: cardiac output; PVR: pulmonary vascular resistance; *dP*/*dt* max: right ventricular maximal isovolumic rate of development of ventricular pressure; *dP*/*dt* min: right ventricular minimal isovolumic rate of development of ventricular pressure. Other abbreviations are in text. *∗* explains *P* < 0.05 for comparison with SU_F; # explains *P* < 0.05 for comparison with RNU_M; § explains *P* < 0.05 for comparison with RNU_F.

## Abbreviation

PAH: Pulmonary hypertension.

## References

[1] Shapiro S., Traiger G. L., Turner M., McGoon M. D., Wason P., Barst R. J. (2012). Sex differences in the diagnosis, treatment, and outcome of patients with pulmonary arterial hypertension enrolled in the registry to evaluate early and long-term pulmonary arterial hypertension disease management. *Chest*.

[2] Humbert M., Sitbon O., Chaouat A. (2010). Survival in patients with idiopathic, familial, and anorexigen-associated pulmonary arterial hypertension in the modern management era. *Circulation*.

[3] Tofovic S. P. (2010). Estrogens and development of pulmonary hypertension: interaction of estradiol metabolism and pulmonary vascular disease. *Journal of Cardiovascular Pharmacology*.

[4] Tofovic S. P., Zhang X., Jackson E. K., Dacic S., Petrusevska G. (2006). 2-Methoxyestradiol mediates the protective effects of estradiol in monocrotaline-induced pulmonary hypertension. *Vascular Pharmacology*.

[5] Sweeney L. B., Bogaard H. J., Natarajan R., Kraskauskas D., Voelkel N. F. Female gender protects against angioproliferative pulmonary hypertension induced by VEGF receptor inhibition and hypoxic exposure.

[6] Taraseviciene-Stewart L., Nicolls M. R., Kraskauskas D. (2007). Absence of T cells confers increased pulmonary arterial hypertension and vascular remodeling. *American Journal of Respiratory and Critical Care Medicine*.

[7] Rondelet B., Kerbaul F., Motte S. (2003). Bosentan for the prevention of overcirculation-induced experimental pulmonary arterial hypertension. *Circulation*.

[8] Paulin R., Michelakis E. D. (2012). The estrogen puzzle in pulmonary arterial hypertension. *Circulation*.

[9] Mair K. M., Johansen A. K. Z., Wright A. F., Wallace E., Maclean M. R. (2014). Pulmonary arterial hypertension: basis of sex differences in incidence and treatment response. *British Journal of Pharmacology*.

[10] Gerber H.-P., McMurtrey A., Kowalski J. (1998). Vascular endothelial growth factor regulates endothelial cell survival through the phosphatidylinositol 3′-kinase/Akt signal transduction pathway: requirement for Flk-1/KDR activation. *The Journal of Biological Chemistry*.

[11] Jacobs W., van de Veerdonk M. C., Trip P. (2014). The right ventricle explains sex differences in survival in idiopathic pulmonary arterial hypertension. *Chest*.

